# 16p13.11 microdeletion/microduplication in fetuses: investigation of associated ultrasound phenotypes, genetic anomalies, and pregnancy outcome follow-up

**DOI:** 10.1186/s12884-022-05267-w

**Published:** 2022-12-07

**Authors:** Meiying Cai, Yanting Que, Xuemei Chen, Yuqing Chen, Bin Liang, Hailong Huang, Liangpu Xu, Na Lin

**Affiliations:** 1 Medical Genetic Diagnosis and Therapy Center, Fujian Maternity and Child Health Hospital College of Clinical Medicine for Obstetrics & Gynecology and Pediatrics, Fujian Medical University, Fujian Key Laboratory for Prenatal Diagnosis and Birth Defect, Fuzhou, China; 2 College of Clinical Medicine for Obstetrics & Gynecology and Pediatrics, Fujian Medical University, Fuzhou, China

**Keywords:** 16p13.11, Nuchal translucency, SNP-array, Phenotypic characteristics, microdeletion/microduplication

## Abstract

**Objectives:**

16p13.11 microdeletion/microduplication are rare genetic diseases with incomplete penetrance, most of which have been reported in adults and children, with ultrasound phenotyping in fetuses rarely described. Here, we have analyzed prenatal ultrasound phenotypic characteristics associated with 16p13.11 microdeletion/microduplication, in order to improve the understanding, diagnosis and monitoring of this disease in the fetus.

**Methods:**

A total of 9000 pregnant women who underwent invasive prenatal diagnosis for karyotyping and SNP-array were retrospectively analyzed in tertiary referral institutions from October 2016 to January 2022.

**Results:**

SNP-array revealed that 20 fetuses had copy number variation (CNV) in the 16p13.11 region, out of which 5 had 16p13.11 microdeletion and the rest showed microduplication, along with different ultrasound phenotypes. Furthermore, 4/20 cases demonstrated structural abnormalities, while the remaining 16 cases were atypical in ultrasound. Taken together, 16p13.1 microdeletion was closely related to thickened nuchal translucency, while 16p13.11 microduplication was more closely associated with echogenic bowel. Only 5/15 fetuses were verified by pedigree, with one case of 16p13.11 microdeletion being *de novo*, and the other cases of 16p13.11 microduplication were inherited from one parent. In 4/20 cases, the pregnancy was terminated. Except for one case with short stature and another one who underwent lung cystadenoma surgery, no abnormalities were reported in the other cases during follow-up.

**Conclusion:**

Fetuses with 16p13.11 microdeletion/microduplication had no characteristic phenotype of intrauterine ultrasound and was in good health after birth, thus providing a reference for the perinatal management of such cases.

## Background

The short arm of chromosome 16 is rich in repeats, including more than 10% of its euchromatin. This special structure makes chromosome 16 a hot spot for replication errors in the human genome, which eventually leads to the occurrence of many microdeletion and microduplication syndromes, especially the 16p13.11 region [1–3]. Previous studies have provided evidence of a strong association between neurodevelopmental disorders and the 16p13.11 locus, although the genes involved within this locus have not been identified [4]. Both microdeletions and microduplications of 16p13.11 may contribute to neuropsychological symptoms, suggesting that this locus harbors dose-sensitive genes that may play a key role in brain development [5].

Previous studies on abnormal copy number variation (CNV) in patients with mental retardation and multiple malformations have shown that the symptoms of some patients may be related to 16p13.11 microdeletion/microduplication [6–8]. It was reported that in addition to microcephaly, developmental delay and a series of mental disorders, patients with 16p13.11 microdeletion also reported a series of epilepsy, while those with 16p13.11 microduplication showed mental retardation, autism, epilepsy and deformative features [9, 10]. These features are more related to adolescent and adult schizophrenia [11], with most reports documenting this clinical phenotype in adults and children. However, studies on fetal ultrasound phenotyping and pathogenesis are still warranted. The application of chromosomal microarray has demonstrated the high-throughput screening of CNV in patients, and also discovered several new genomic rearrangements caused by LCRs. In this study, we used single nucleotide polymorphism array (SNP-array) detected 16p13.11 microdeletion/microduplication in fetus, along with analysis of ultrasound phenotypes, genetic testing results, and pregnancy outcomes, in order to improve the understanding, diagnosis and monitoring of these genetic abnormalities in the fetus.

## Methods

### Patient recruitment

9000 pregnant women who underwent invasive prenatal diagnosis for karyotyping and SNP-array were retrospectively analyzed in tertiary referral institutions from October 2016 to January 2022. The average age of pregnant women was 28.2 years (range: 18–47 years); The mean gestational age was 23.4 weeks (range: 16-38 weeks). Transabdominal amniocentesis or umbilical cord blood puncture were selected according to the gestational age of the pregnant women. All pregnant women received genetic counseling and signed informed consent prior to invasive diagnosis. This study was approved by the Medical Ethics Committee of Fujian Provincial Maternal and Child Health Hospital (2,014,042).

### Traditional karyotype analysis

Samples of amniotic fluid or umbilical cord blood were collected and cultured in 1640 medium (Hangzhou Bosheng Company) in a 5% carbon dioxide incubator at 37℃. Cells were harvested from cord blood samples after 3 days of culture and from amniotic fluid samples after 8 days of culture. Colchicine was added 1 h before harvest to maintain the cells in the mitotic metaphase. After harvesting, the cells were prepared, and G-banding was performed. Finally, the karyotype was collected by GSL-120 automatic chromosome scanning platform, followed by calculation and analysis. According to the International Nomenclature System of Human Cytogenetics (ISCN 2016), 40 karyotypes were counted in each case and 5 were analyzed. In case of abnormalities, 20 karyotypes were added for counting and analysis.

### SNP-array

DNA was extracted from fetal tissues using a genome-wide DNA extraction kit (Qiagen, Germany). Cytoscan750k chip (Affymetrix, USA) was used to hybridize the whole genome. The process included DNA digestion, PCR, followed by PCR product purification, fragmentation, labeling, hybridization, washing, staining, and scanning. Affymetrix Chromosome Analysis Suite (ChAS) 3.2 was used for data Analysis. SNP-array results were further analyzed to determine the nature of CNV according to the relevant databases. The following databases are mainly referred to: International public DGV benign variation database (http://projects. The tcag. Ca/variation), international public DECIPHER pathological variation database (HTTS: / / decinher. Sanger. Ac. UK /), Online Human Mendelian Genetic Database OMIM (http://www.omim.org), International federation of cell gene chip standardized ISCA (https://www.iscaconsortium.org/) and the global Affymetrix pathological Shared database user CAGdb (http://www.cagdb.org/), CHD Wiki, NCBI PubMed, etc. According to the guidelines for medical Genetics in the United States [12], CNV should be divided into pathogenic, likely pathogenic, copy number variants with uncertain clinical significance (VUS), likely benign and benign.

### Follow-up of obstetric outcomes

All fetuses were examined regularly, and the condition of the fetus was observed by dynamic ultrasound. Pregnancy outcomes and neonates were followed up. After birth, the parents of the surviving infants were followed up by telephone to evaluate the physical growth and neurobehavioral development of the child.

## Results

### SNP-array results for the fetus

**Fig. 1:**
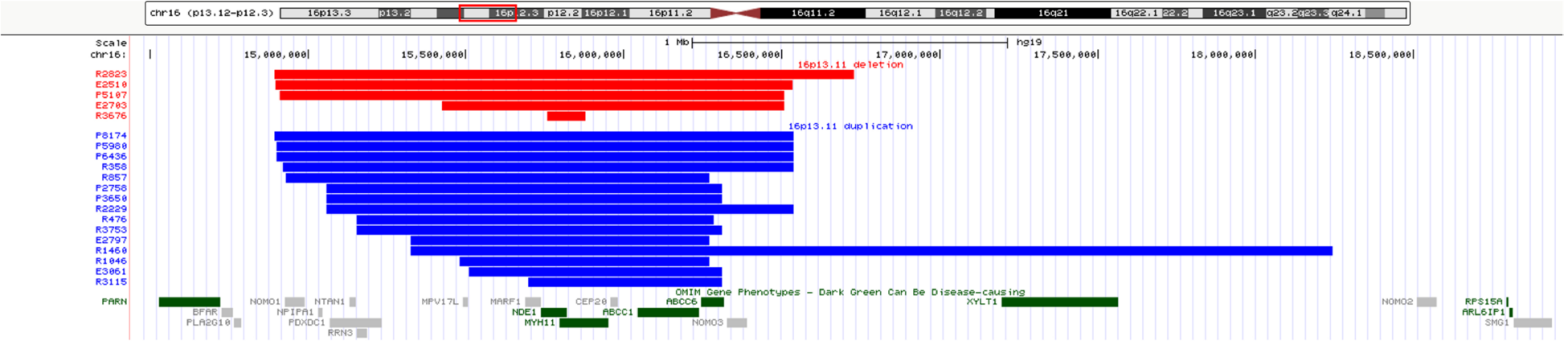
16p13.11 microdeletion/microduplication detected using SNP-array. SNP-array revealed 16p13.11 microdeletion in fetus E2510, E2703, P5107, R2823 and R3676, 16p13.11 microduplication in fetus E2797, E3061, P2758, P3650, P5980, P6436, P8174, R358, R476, R857, R1046, R1460, R2229, R3115, and R3753

SNP-array revealed that 20 fetuses had differences in CNV in the 16p13.11 region (Table 1). Five fetuses had reduced CNV in the 16p13.11 region, involving fragments ranging from 0.12 Mb to 1.8 Mb and containing 11 to 45 OMIM genes (Fig. 1). Fifteen fetuses had increased CNV in the 16p13.11 region, involving fragments ranging in size from 0.60 Mb to 2.92 Mb and containing 5 to 19 OMIM genes (Fig. 1).

**Table 1:** SNP-array of 20 fetuses with 16p13.11 microdeletion/microduplication

Case	SNP-array	OMIM gene	CNV	Size(Mb)	Inheritance
E2510	arr[hg19]16p13.11(14,897,401-16,534,031)x1	11	Loss	1.6	Refused
E2703	arr[hg19]16p13.11(15,422,960-16,508,123)x1	34	Loss	1.0	*denovo*
P5107	arr[hg19]16p13.11(14,910,158-16,508,123)x1	45	Loss	1.6	Refused
R2823	arr[hg19]16p13.11(14892975_16730375)x1	11	Loss	1.8	Refused
R3676	arr[hg19]16p13.11(15756822_15877444)x1	11	Loss	0.12	Refused
E2797	arr[hg19]16p13.11(15,325,072-16,272,403)x3	11	Gain	0.92	Refused
E3061	arr[hg19]16p13.11(15,510,512-16,309,046)x3	5	Gain	0.78	Refused
P2758	arr[hg19]16p13.11(15,058,820-16,309,046)x3	8	Gain	1.25	Refused
P3650	arr[hg19]16p13.11(15,058,820-16,309,046)x3	19	Gain	1.25	Paternal
P5980	arr[hg19]16p13.11(14,900,042-16,538,596)x3	11	Gain	1.6	Refused
P6436	arr[hg19]16p13.11(14,900,042-16,508,123)x3	11	Gain	1.6	Refused
P8174	arr[hg19]16p13.11(14,892,975-16,538,596)x3	12	Gain	1.6	Refused
R358	arr[hg19]16p13.11(14,920,864-16,538,596)x3	11	Gain	1.65	Refused
R476	arr[hg19]16p13.11(15,154,356-16,282,869)x3	7	Gain	1.12	Maternal
R857	arr[hg19]16p13.11(14,929,070-16,272,403)x3	10	Gain	1.3	Refused
R1046	arr[hg19]16p13.11(15,481,747-16,272,403)x3	5	Gain	0.77	Paternal
R1460	arr[hg19]16p13.11p12.3(15,325,072-18,242,713)x3	7	Gain	2.92	Refused
R2229	arr[hg19]16p13.11(15,058,820-16,538,596)x3	9	Gain	1.48	Refused
R3115	arr[hg19]16p13.11(15697535_16309046)x3	5	Gain	0.60	Maternal
R3753	arr[hg19]16p13.11(15154356_16309046)x3	5	Gain	1.2	Refused

### Traditional karyotype analysis for the fetus

The traditional karyotype analysis of 20 fetuses with 16p13.11 microdeletion/microduplication was negative.

### Ultrasound phenotype for the fetus

The ultrasound performance of 20 fetuses with 16p13.11 microdeletion/microduplication was variable. Except for 4 fetuses with structural abnormalities (ventricular septal defect, urorectal septal malformation sequence, right renal duplication, and lung cyst adenomatous lesions), the other 16 cases were atypical. Among the 16 fetuses with atypical ultrasonography, the most common feature was abnormal ultrasonic soft indicators, including 4 cases of echogenic bowel, 3 cases of thickened nuchal translucency and 3 cases of ventricle widening (one of them had both echogenic bowel and ventriculomegaly). In fetuses with 16p13.11 microdeletion, 2/5 fetuses had thickened nuchal translucency, and in fetuses with 16p13.11 microduplication, 3/12 fetuses had echogenic bowel. There were 6 cases with no obvious abnormalities on ultrasound examination. The possible reason was prenatal diagnosis, which was performed because of high risk for Down’s syndrome (*n* = 3), one parent was a carrier of balanced translocation (*n* = 2), oradvanced age (*n* = 1). The ultrasonographic details of the 20 fetuses are shown in Table 2.

**Table 2:** Clinical information and ultrasound characteristics of 20 fetuses with 16p13.11 microdeletion/microduplication

Case	Ultrasound characteristics	Pregnancy outcome	Outcome of live‐born
E2510	Ventricular septal defect	Cesarean	Age of 4 years, Boy, Well survivor
E2703	Ventriculomegaly, Enhanced intestinal echo	TP	-
P5107	Thickened nuchal translucency	Cesarean	Age of 3 years, Girl, Short stature, Normal mental development
R2823	Normal(High risk for Down's screening)	TP	-
R3676	Thickened nuchal translucency	Eutocia	Age of 6 months, Boy, Well survivor
E2797	Urorectal septal malformation sequence	TP	-
E3061	Mild tricuspid regurgitation	Eutocia	Age of 3 years, Girl, Well survivor
P2758	Enhanced intestinal echo	Eutocia	Age of 4 years, Boy, Well survivor
P3650	Ventriculomegaly	Eutocia	Age of 4 years, Girl, Well survivor
P5980	Normal(Balanced translocation of chromosomes in the father of the fetus)	Cesarean	Age of 3 years, Boy, Well survivor
P6436	Enhanced intestinal echo	TP	-
P8174	Right renal duplication	Eutocia	Age of 2 years, Girl, Well survivor
R358	Normal(Balanced translocation of chromosomes in pregnant women)	Eutocia	Age of 1.5 years, Girl, Well survivor
R476	Lung cystadenomatous lesions	Eutocia	Age of 1.5 years, Girl, Postnatal lung cystadenoma surgery, Everything else is normal
R857	Normal(High risk for Down's screening)	Eutocia	Age of 4 months, Boy, Well survivor
R1046	Thickened nuchal translucency	Cesarean	Age of 1.3 years, Boy, Well survivor
R1460	Normal(High risk for Down's screening)	Cesarean	Age of 1.2 years, Girl, Well survivor
R2229	Enhanced intestinal echo	Cesarean	Age of 1.1 years, Boy, Well survivor
R3115	Ventriculomegaly	Eutocia	Age of 8 months, Boy, Well survivor
R3753	Normal(advanced maternal age)	Cesarean	Age of 6 months, Boy, Well survivor

*TP* Termination of pregnancy

### Results of SNP-array pedigree analysis

In addition to the 5 fetuses, the parents of the other 15 fetuses refused pedigree verification. Out of the 5 cases, one case of 16p13.11 microdeletion was *denovo*, and the other 4 cases of 16p13.11 microduplication were maternally (*n* = 2) or paternally (*n* = 2) inherited (Table 1).

### Pregnancy outcome

In 20 cases with CNV changes in the 16p13.11 region, the parents of 4 fetuses chose to terminate the pregnancy, while in the other 16 fetuses chose to continue the pregnancy after adequate genetic counseling regarding the possible risks (Table 2). At present, the age range of 16 cases who can be followed up at term delivery is from 4 months to 4 years. Except for one case who was found to have short stature and one case who underwent lung cystadenoma surgery, no abnormalities have been detected in the other cases during follow-up via telephone (Table 2).

## Discussion

Region 16p13.11 is a dose-sensitive region whose microdeletions or microduplications can lead to a variety of clinical phenotypes [13, 14]. 16p13.11 microdeletions are well defined in clinical practice. However, 16p13.11 microduplication is a newly discovered syndrome whose molecular mechanism, candidate genes, and pathogenesis remain unclear [15]. In this study, CNV analysis was performed on over 9000 fetuses undergoing prenatal diagnosis using SNP-array. It was seen that 20 fetuses had CNV changes in the 16p13.11 region, including 5 cases with 16p13.11 microdeletion and 15 cases with 16p13.11 microduplication.

It has been reported that the size of 16p13.11 microdeletion region ranged from 0.8 to 3.3 Mb [13]. In this study, there were 5 cases of 16p13.11 microdeletion involving fragments of approximately 0.12 Mb to 1.8 Mb in size. The region of the 16p13.11 microdeletion was mostly within the range reported in earlier studies, except for one case where it was only 0.12 Mb. A large number of clinical studies have shown that 16p13.11 microdeletion is strongly correlated with a variety of neurological disorders, such as intellectual disability, epilepsy, schizophrenia, etc. Heinzen et al. [16] conducted genome-wide CNV analysis on patients with epilepsy syndrome and found that 23 of them carried 16p13.11 microdeletions, with clinical manifestations of partial epilepsy, childhood amnesic epilepsy or juvenile amnesic epilepsy. Hannes et al. [17] conducted comparative genomic hybridization screening on patients with intellectual disability and multiple malformations and found that the presence of 16p13.11 microdeletion could increase these neurological problems. Previous studies have reported that patients with 16p13.11 microdeletion have diverse clinical phenotypes, which can manifest as intellectual disability, autism, epilepsy, microcephaly, short stature and other abnormalities, while some patients may have no obvious clinical abnormalities [18]. However, there are few reports on 16p13.11 microdeletion in fetuses. Paciorkowski et al. [19] reported on two fetuses with severe microcephaly, agenesis of the corpus callosum, scalp rugae, and a fetal brain disruption like phenotype with deletions in the 16p13.11 region. Similarly, in another study, a fetus with 16p13.11 microdeletion with post hemorrhagic hydrocephalus with marked ventriculomegaly, cortical thinning, hypoplastic falx cerebri, cleft lip on right, two preauricular skin tags on right, and cleft T1 and T3 vertebral bodies was reported [17]. However, in our study, the ultrasound phenotype of a fetus with 16p13.11 microdeletion had ventriculomegaly, which was consistent with the previous literature survey. Coello-Cahuao et al. [20] found that 16p13.11 microdeletion was reported in 2.5% of the cohorts of fetuses with thickened nuchal translucency. In this study, 2/5 fetuses with 16p13.1 microdeletion had this defect, indicating a close relationship of 16p13.1 microdeletion with thickened nuchal translucency. The ultrasound characteristics of 2 fetuses with 16p13.11 microdeletion were ventricular septal defect and echogenic bowel, which have not been reported so far. At the same time, the ultrasound phenotype of one fetus was normal. The cause of phenotypic diversity in patients with 16p13.11 microdeletion is still unclear and detailed analysis is warranted [21].

16p13.11 microduplication has been linked to autism and neuropsychiatric disorders, including schizophrenia, attention deficit hyperactivity disorder, and intellectual disability [11, 22–25]. In fact, the influence of 16p13.11 microduplication is not without controversy, and some studies have reported it as a rare benign variant [26]. However, two studies involving large case-control cohorts consistently reported a predisposition of 16p13.11 microduplication to autism spectrum disorder (ASD) and other types of neurodevelopmental disorders, with statistically significant results [11, 27]. Khattabi et al. [28] reported that the most common clinical features of 16p13.11 microduplication were developmental delay, intellectual deficiency or ASD. Cardiac abnormalities, especially aortic abnormalities, may also occur in some patients [29]. Few studies have also reported that 16p13.11 microduplication is a known susceptibility locus for neurocognitive diseases, with incomplete externality and performance differences, and its penetrance was approximately 7–8% [2, 30, 31]. The clinical phenotype of patients varies greatly, which can be manifested as autism spectrum disorder, learning difficulties, brain MRI abnormalities, heart malformation and other abnormalities. So far, studies on fetus with 16p13.11 microduplication have been limited, and only a few of them report about malformation findings. Dąbkowska et al. [32] reported microduplication 16p13.11 in one fetus with prenatally diagnosed cephalocele. In this study, the ultrasound phenotype of 15 fetuses with 16p13.11 microduplication was studied. Only 3 fetuses had structural abnormalities (urorectal septum malformation sequence sign, right renal duplicates and lung cyst adenomatous lesions), and the rest were atypical. Among the 12 fetuses with atypical ultrasonic phenotype, the most common feature was echogenic bowel (*n* = 3), followed by ventriculomegaly (*n* = 2), thickened nuchal translucency (*n* = 1) and mild tricuspid regurgitation (*n* = 1), and normal ultrasonic phenotype (*n* = 5). From these findings, it can be concluded that 16p13.11 microduplication is most closely associated with echogenic bowel. However, the molecular basis of how 16p13.11 microduplication leads to disease remains unclear, which requires further research on the pathogenic mechanism [33].

Studies have reported that pathogenic 16p13.11 microdeletion/microduplication is inherited from the normal phenotype of parents or denovo, while some patients may have no obvious clinical abnormalities [2]. As a result of genetic heterogeneity, 16p13.11 microdeletion/microduplication result in a clinical phenotype that shows explicit differences in expression. It has been reported [18] that the haplodose deficiency effect score of 16p13.11 region was 3, with a penetrance of about 13.1%, and the clinical phenotypes of patients were diverse [18] while the triple dose sensitive effect score of 16p13.11 region was 2, with a penetrance of about 7–8%, indicating a large difference in clinical phenotypes [28, 34]. Among the 5/20 fetuses verified by pedigree in this study, one case with 16p13.11 microdeletion was a *denovo*, and the ultrasound phenotype of the fetus was ventriculomegaly and echogenic bowel. After genetic counseling, the parents of the fetus chose to terminate the pregnancy. The other 4 cases with 16p13.11 microduplication were inherited from parents with normal phenotypes. After genetic counseling, the parents of these 4 fetuses chose to continue the pregnancy, and no abnormalities were found in the neonates during follow-up after birth. Only one fetus underwent lung cystadenoma surgery after birth and was followed up to the age of 1.5 years with normal height, weight and intelligence. The parents of 15 fetuses refused pedigree verification, among which the parents of 3 fetuses chose to terminate pregnancy after genetic counseling, and the other 12 chose to continue the pregnancy. During follow-up of the 12 fetuses after birth, no abnormalities were found in the remaining 11 cases, except for one case exhibiting short stature. In conclusion, it can be suggested that when the dose of 16p13.11 gene is found to be changed in the fetus, the pregnancy should not be terminated blindly, and comprehensive judgment should be made in all aspects, such as combining fetal ultrasound phenotype and family analysis.

This study has a few limitations. First, the small sample size, with only 20 cases of fetus being detected with 16p13.11 microdeletion/microduplication. Second, single gene mutation was not detected in the method. As a new genetic detection technology, next-generation sequencing is used to detect single gene mutations and copy number variations, which may provide more comprehensive prenatal genetic diagnosis for fetuses with 16p13.11 gene dose changes and better assessment for fetal prognosis. Third, the longest follow-up cases of this study could be tracked only up to 4 years of age, with the literature reporting clinical phenotype of 16p13.11 microdeletion/microduplication mostly in adults and children [10, 35, 36]. Therefore, it is necessary to follow up these cases for a long time in the future to observe the changes in the clinical phenotype, if any, for better clinical intervention. In future studies, more cases should be included, so that a single gene can be tested at the same time, for better perinatal management and clinical guidance after birth.

## Conclusion

Taken together, with the resolution of conventional karyotype analysis being limited for the detection of microdeletion/microduplication, SNP- array can effectively diagnosis the 16p13.11 microdeletion/microduplication. We found that 16p13.1 microdeletion was closely related to thickened nuchal translucency, however 16p13.11 microduplication was more closely associated with echogenic bowel. At the same time, we conducted ultrasound phenotype analysis, pregnancy outcome follow-up and postnatal follow-up of the fetuses with 16p13.11 microdeletion/microduplication, and found that these fetuses were in good health after birth, thereby providing a reference for the perinatal management of these fetuses.

## Data Availability

Data and materials are available on reasonable request. The data and materials that support the findings of this study are available from the corresponding author on reasonable request.
